# Effects of different sensory integration tasks on the biomechanical characteristics of the lower limb during walking in patients with patellofemoral pain

**DOI:** 10.3389/fbioe.2024.1441027

**Published:** 2024-08-27

**Authors:** Fan Ting, Zhang Zeyi

**Affiliations:** ^1^ Shanghai Zhuoyue Ruixin Digital Technology Company limited, Shanghai, China; ^2^ School of Physical Education and Health Care, East China Normal University, Shanghai, China; ^3^ Key Laboratory of Adolescent Health Assessment and Exercise Intervention of Ministry of Education, East China Normal University, Shanghai, China

**Keywords:** sensory integration task, patellofemoral pain, biomechanical of the lower limb, gait, patellofemoral joint stress

## Abstract

**Purpose:**

This study aimed to analyze the biomechanical characteristics of the lower limb in patients with patellofemoral pain (PFP) while walking under different sensory integration tasks and elucidate the relationship between these biomechanical characteristics and patellofemoral joint stress (PFJS). Our study’s findings may provide insights which could help to establish new approaches to treat and prevent PFP.

**Method:**

Overall, 28 male university students presenting with PFP were enrolled in this study. The kinematic and kinetic data of the participants during walking were collected. The effects of different sensory integration tasks including baseline (BL), Tactile integration task (TIT), listening integration task (LIT), visual integration task (VIT) on the biomechanical characteristics of the lower limb were examined using a One-way repeated measures ANOVA. The relationship between the aforementioned biomechanical characteristics and PFJS was investigated using Pearson correlation analysis.

**Results:**

The increased hip flexion angle (*P* = 0.016), increased knee extension moment (*P* = 0.047), decreased step length (*P* < 0.001), decreased knee flexion angle (*P* = 0.010), and decreased cadence (*P* < 0.001) exhibited by patients with PFP while performing a VIT were associated with increased patellofemoral joint stress. The reduced cadence (*P* < 0.050) achieved by patients with PFP when performing LIT were associated with increased patellofemoral joint stress.

**Conclusion:**

VIT significantly influenced lower limb movement patterns during walking in patients with PFP. Specifically, the increased hip flexion angle, increased knee extension moment, decreased knee flexion angle, and decreased cadence resulting from this task may have increased PFJS and may have contributed to the recurrence of PFP. Similarly, patients with PFP often demonstrate a reduction in cadence when exposed to TIT and LIT. This may be the main trigger for increased PFJS under TIT and LIT.

## 1 Introduction

Patellofemoral pain (PFP) is one of the most prevalent overuse condition of the lower limb, with an annual prevalence rate of 23% (Smith et al., 2018). Of those affected, 70%–90% experience a recurrence of pain, which may increase the risk of developing patellofemoral arthritis (Stathopulu Baildam, 2003). Statistical data indicates that the annual *per capita* medical cost of patients with PFP can be as high as 300 euros (Tan et al., 2010). This demonstrates that PFP not only significantly impacts the quality of life of patients, but also generates high medical and socio-economic costs. Consequently, there is an urgent need to identify effective strategies for preventing the recurrence of pain in patients with PFP (Tan et al., 2010).

A review of relevant literature showed that PFP is frequently associated with functional activities, such as running, deep squatting, and walking up and down a flight of stairs. This is because patients with PFP often exhibit greater patellofemoral joint stress (PFJS) when completing these exercises. The PFJS is the patellofemoral joint force (PFJF) per unit area of patella-femur contact. An increase in PFJS will result in an elevated immediate loading rate of the patellofemoral cartilage, which will subsequently impact the internal homeostasis of the patellofemoral joint, potentially leading to the onset of pain episodes (Kedroff et al., 2019). Further, a recent study identified cumulative loading of the patellofemoral joint as a significant contributing factor to PFP (Petersen et al., 2015). Although daily physical activities, such as walking, do not typically result in acute PFP, due to the high frequency of walking in daily life, incorrect movement patterns can lead to an increased cumulative load on the patellofemoral joint, which can disrupt the constant physiological environment within the patellofemoral joint (Willy and Meira, 2016), thereby increasing the risk of PFP (Song et al., 2023). Consequently, a number of studies have investigated the diagnosis and monitoring of gait patterns in patients with PFP with the objective of identifying gait patterns that could be modified with intervention, with the ultimate goal of reducing the symptoms or risk of developing PFP.

Related research indicates that cognitive perturbation while walking is highly variable, including activities such as observing the road, conversing with others, and carrying handheld objects. These distractions have been shown to influence gait patterns (Abdallat et al., 2020). Stöckel and Mau-Moeller (2020) observed that playing games on a smartphone while walking resulted in a 26.8% reduction in step speed and a 60.2% increase in gait variability. A reduction in stride speed may be attributed to a decline in either stride frequency or stride length. A diminution in stride frequency may result in an extended single-support phase during ambulation, a prolonged loading period at the knee, and ultimately, an augmented loading of the knee, which may elevate the risk of recurrent patellofemoral joint discomfort (Heiderscheit et al., 2011; Van Hooren et al., 2024). Martín-Martínez et al. (2020) examined the impact of cognitive perturbation on the gait pattern of female individuals. The results indicated that cognitive perturbation leads to an increase in step length and a decrease in cadence, this gait characteristic results in an increased knee flexion angle, which in turn leads to an elevated internal loading of the knee. This ultimately causes an increase in PFJS (Lenhart et al., 2014). Conclusions similar to those presented here were reached by Bonacci et al. (2018), who found that reducing the step frequency during running in patients with PFP resulted in significantly higher patellofemoral joint reaction forces and PFJS. This is due to the fact that a reduction in stride frequency and an increase in stride length result in a greater percentage of energy being absorbed by the knee joint, which in turn leads to an increase in patellofemoral joint contact pressure and an elevation of PFJS (Lenhart et al., 2015). However, Júnior et al. (2017) investigation into the impact of oral cognitive perturbation on gait patterns revealed that cognitive perturbation can result in a reduction in step length and an increase in single support time. A review of the literature reveals that there is no consensus among researchers regarding the effects of cognitive perturbation on gait patterns. The discrepancies in findings can be attributed to the heterogeneity of the cognitive perturbation employed in the relevant studies. Different cognitive perturbations can elicit stimulation and interference in the human body through distinct sensory modalities, including vision, listening, and touch, among others. Initial research has demonstrated that the interaction between different sensory inputs and the brain can be highly variable, with potential implications for gait patterns (King and Walker, 2012).

This study aims to investigate the impact of different sensory integration tasks, including baseline (BL), tactile integration task (TIT), listening integration task (LIT), visual integration task (VIT) on the gait patterns of patients with PFP. Additionally, the study seeks to elucidate the relationship between these gait patterns and PFJS to identify biomechanical factors associated with PFP. Finally, the study aims to provide theoretical insights for the rehabilitation training of patients with PFP in the future. Based on previous literature and the objective of this study, the following research hypotheses are tested in this study: The impact of different sensory integration tasks on gait characteristics varies. The VIT exerts the most significant influence, with alterations in gait characteristics resulting from this task potentially leading to an increase in the PFJS.

## 2 Materials and methods

### 2.1 Participants

It has been observed that the prevalence of PFP in women is approximately twice that in men (Boling et al., 2010). Consequently, the majority of current research on PFP is focused on young female patients. However, it is notable that the annual prevalence of PFP in young men has also reached 15.5% (Smith et al., 2018), and this group should not be overlooked. Further research is required to elucidate the underlying causes of PFP recurrence in the male population. It was for this reason that male patients with PFP (16–25 years) were selected for inclusion in the study. Prior to the commencement of the experiment, the participants’ pain level, pain duration and level of knee function were evaluated. The details are as follows: the VAS score of 0 indicated the absence of pain symptoms, whereas a score of 10 indicated the presence of the most severe pain symptoms. The AKPS scale ranged from 0 to 100, with lower scores indicating a poorer level of knee function. The inclusion criteria were as follows (Biabanimoghadam et al., 2016): (1) the presence of retro-patellar or peripatellar pain during at least two of the following activities: running, jumping, sedentary activities, walking up and down a flight of stairs, resisted knee extension, and single-leg squatting; (2) the presence of pain for at least 3 months that was not related to direct trauma; and (3) the pain symptoms or the degree of pain was related to the amount of running or the intensity of the exercise. (4) The visual analog scale (VAS) score was less than 3. In this study, participants were required to be free of pain at the time of the test. The level of pain in the patellofemoral joint was scored on the VAS scale, and a score of less than 3 was considered indicative of the absence of pain. The exclusion criteria included the following conditions: (1) the presence of other knee pathologies, such as rheumatoid arthritis, osteoarthritis, and injuries to the patellar tendon, quadriceps tendon, meniscus, or ligaments; (2) the presence of a patellar subluxation or dislocation; and (3) the presence of surgery to the lower extremity within 1 year. A single professional tester conducted the screening, which involved 28 participants, aged 21.5 ± 2.3 years, with a height of 177.6 ± 3.8 cm, a weight of 73.2 ± 6.9 kg, VAS score of 0.5 ± 0.4, a pain duration of 104.6 ± 18.8 days, and AKPS score of 87.8 ± 10.5. Prior to undertaking formal testing, the tester underwent rigorous clinical training. The trainer is a professor of sports biomechanics at East China Normal University. These participants were included according to the aforementioned criteria. Consent for the publication of personal data in this study was obtained from the participants. The study was performed in accordance with the ethical standards of the Declaration of Helsinki given ethics approval was obtained from the Ethics Committee of East China Normal University on 22 May 2024.

### 2.2 Test apparatus

#### 2.2.1 Motion capture system

The kinematic data in this experiment was acquired through 12 Vicon Vero2.2 infrared cameras situated at a height of 2.5 m. These cameras were used to capture and record the movement trajectory of infrared reflective markers, with a sampling frequency of 100 Hz. This data was then combined with a motion capture system to obtain the 3D coordinates of the markers and calculate the 3D kinematic data during walking.

#### 2.2.2 Three-dimensional force plate

The kinetic data for this experiment was collected using two Kistler 3D force plates (Kistler Inc., Switzerland) measuring 80 cm × 60 cm and a sampling frequency of 1,000 Hz. These force plates were designed to capture the 3D kinetic data of the participants as they walked.

### 2.3 Experimental design and testing procedures

**FIGURE 1:**
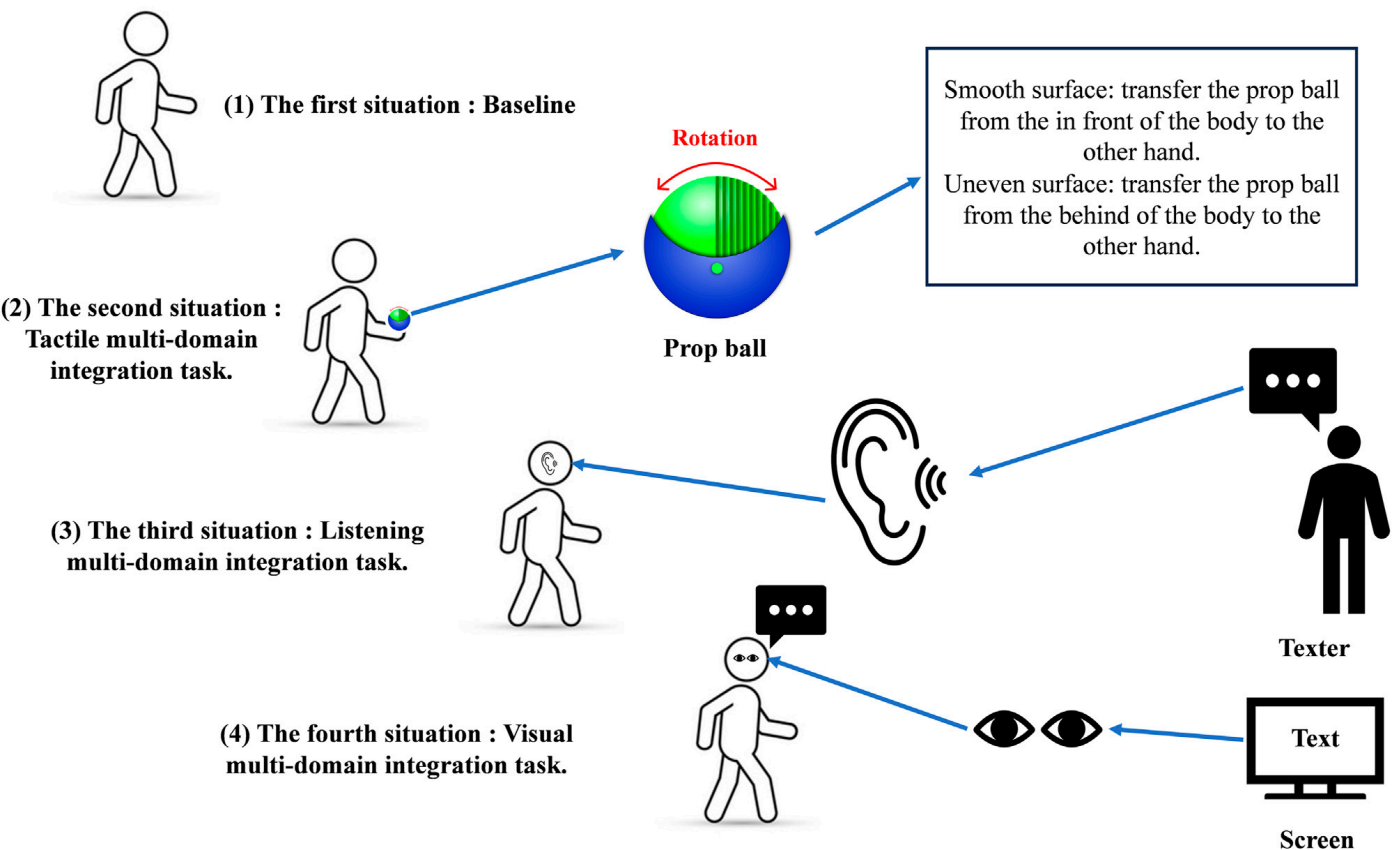
Schematic of the prop ball and the four sensory tasks.

Prior to the commencement of the test, the participants were provided with uniform footwear (Nike WINFLO 10) and shorts. Thereafter, the participants were furnished with reflective markers, which was affixed in accordance with the plug-in-gait model, specifically as follows: Left ASIS, Right ASIS, Left PSIS, Right PSIS, Left knee, Right knee, Left thigh, Right thigh, Left ankle, Right ankle, Left tibial, Right tibial, Left toe, Right toe, Left heel, Right heel. Two three-dimensional force platforms were embedded in the middle of the walkway to collect kinetic data during walking. Prior to the commencement of the formal experiment, the static calibration movements of the participants were recorded. These included the feet being positioned shoulder-width apart, the trunk being maintained in a straight position, the arms being raised sideways with the palms facing downwards, and the eyes directed forward. At the outset of the formal experiment, the participants were requested to complete a series of walking maneuvers on a 10 × 2 m trail at a self-selected comfortable speed, with the same movement pattern as that employed during daily walking. This was done while they performed a series of sensory integration tasks, the order of which was randomly selected by the computer. The four tasks were as follows: (1) BL, which was to be observed without any distractions. (2) TIT, which required subjects to hold a prop ball in one hand, rotated it with the fingers, and then used the fingers to halt the rotation while palpating the surface of the ball to ascertain whether it was smooth or uneven. Once the rotation of the ball has ceased, if the fingers were in contact with a smooth surface, the subject would transfer the prop ball from the in front of the body to the other hand. In the event that the contact surface was uneven, the subject would transfer the prop ball from the behind of the body to the other hand. And the frequently of stopping the ball and shifting it to the opposite hand was the once every 3 s during the 10 m walk. (3) LIT, in which a text was read aloud by the same tester, followed by a repetition of the text by the participant, with at least 90% text overlap. The participants were instructed to walk to a distance of 7 m and commence repeating the textual content. The LIT was designed to simulate the listening component of a conversation. Repeating 90% of the textual content was intended to ensure that the participant had listened carefully and identified the relevant information. (4) VIT required participants to view a text (presented on a 15-inch electronic screen) and read it aloud in order to ensure that it was correctly identified during the test. The task focused on simulating the ecological context of reading while walking (Figure 1). A total of three valid gait data sets were collected in the experiment. In order to avoid any potential cognitive fatigue in the participants, the interval between each test was set at 3 min.

### 2.4 Data extraction and preparation

#### 2.4.1 Kinematics

**FIGURE 2:**
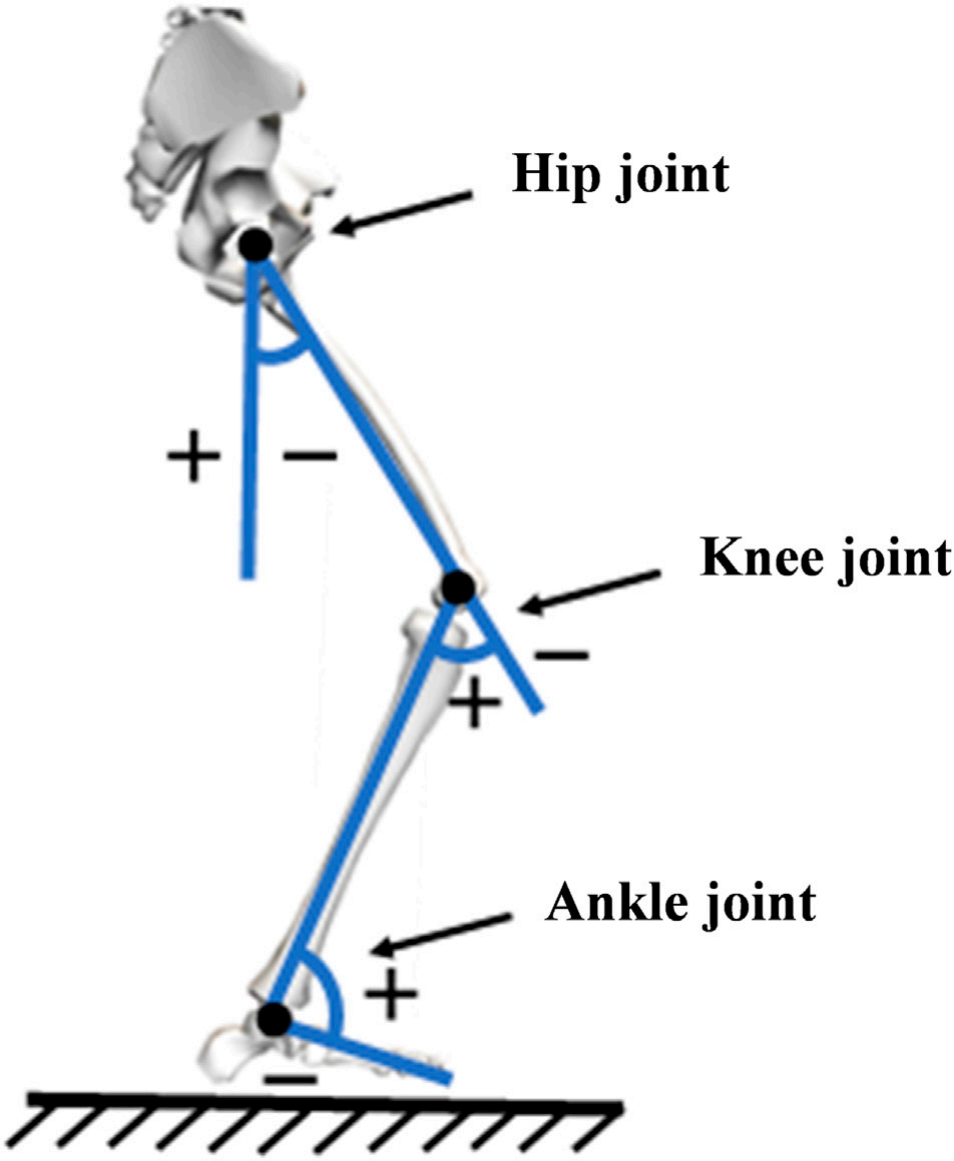
Schematic diagram of the calculation method for joint angle.

The objective of this study was to analyze the kinematic data at the time of maximum PFJS during the single-support period of the affected leg. The kinematic data were calculated using Vicon vero2.2 software (Vicon Inc., UK). The original markers trajectory was initially low-pass filtered using a 10 Hz fourth-order Butterworth filter. Thereafter, the joint angles in the sagittal, frontal, and horizontal planes of the hip, knee, and ankle joints at the moment of maximum PFJS of the walking leg were calculated by establishing the transformation matrix, calculating the joint center, and establishing the local coordinate system of the link (Figure 2). The inverse dynamics method was used to calculate the three dimensions net joint moment (Bresler and Frankel, 1950). The joint power is defined as the product of the net joint moment and the joint angular velocity (Elmer et al., 2011). Subsequently, gait parameters were derived. Step length was defined as the longitudinal distance between the left and right heels. Step width was the medial-lateral distance between the left and right feet following the ground. Cadence is the number of steps taken per minute. The foot progress angle was defined as the angle between the supporting foot through the plantar centerline and the longitudinal axis of the forward direction.

#### 2.4.2 Kinetics

The objective of this study was to analyze the kinetic data of the affected leg-support period in patients with PFP. The raw ground reaction force (GRF) data were subjected to low-pass filtering using a 50-Hz fourth-order Butterworth filter. The affected leg-support period was determined by identifying the moment of touchdown as the point at which the GRF value exceeded 20 N, and the moment of departure from the ground as the point at which the GRF value fell below 20 N.

The patellofemoral joint force is the force between the patella and the femur due to contact. The PFJS is the patellofemoral joint force per unit area of patella-femur contact. Finally, the quadriceps muscle force is the force generated by the quadriceps muscle during contraction and extension (Sherman et al., 2014). The PFJS was calculated in accordance with the following methodology (Bressel, 2001; Vannatta and Kernozek, 2015):

$$F_{Q}\left(\theta_{i}\right)=M_{EXT}\left(\theta_{i}\right)/L_{A}\left(\theta_{i}\right)$$

*F*_*Q*_ represents quadriceps muscle force (N), *M*_*EXT*_ denotes knee-extension moment (N·m), *L*_*A*_ signifies effective quadriceps muscle arm (cm), and *θi* denotes the knee flexion-extension angle (°) of the *i*th frame.

This study posits that a positive sagittal plane net moment of the knee during walking represents the knee extension moment, as follow:

$$M_{EXT}=M_{NET}$$

In the formula, M_NET_ represents the sagittal plane knee net moment (N·m). The effective muscle force arm of the quadriceps muscle is a function of the sagittal plane knee joint angle *θ* (°):

$$L_{A}=\left\{\begin{array}{c} 0.036\theta +3.0\left(0{}^{\circ}\leq \theta \;<\;30{}^{\circ}\right)\\ -0.043\theta +5.4\left(30{}^{\circ}\leq \theta \;<\;60{}^{\circ}\right)\\ -0.027\theta +4.3\left(60{}^{\circ}\leq \theta \;<\;90{}^{\circ}\right)\\ 2.0\left(90{}^{\circ}\leq \theta \right) \end{array}\right\}$$

Patellofemoral joint force calculation:

$$F_{PF}=2F_{Q}\sin\left(\beta /2\right)$$

Among others:

$$\beta =30.46+0.53\left(\theta \right)$$

**FIGURE 3:**
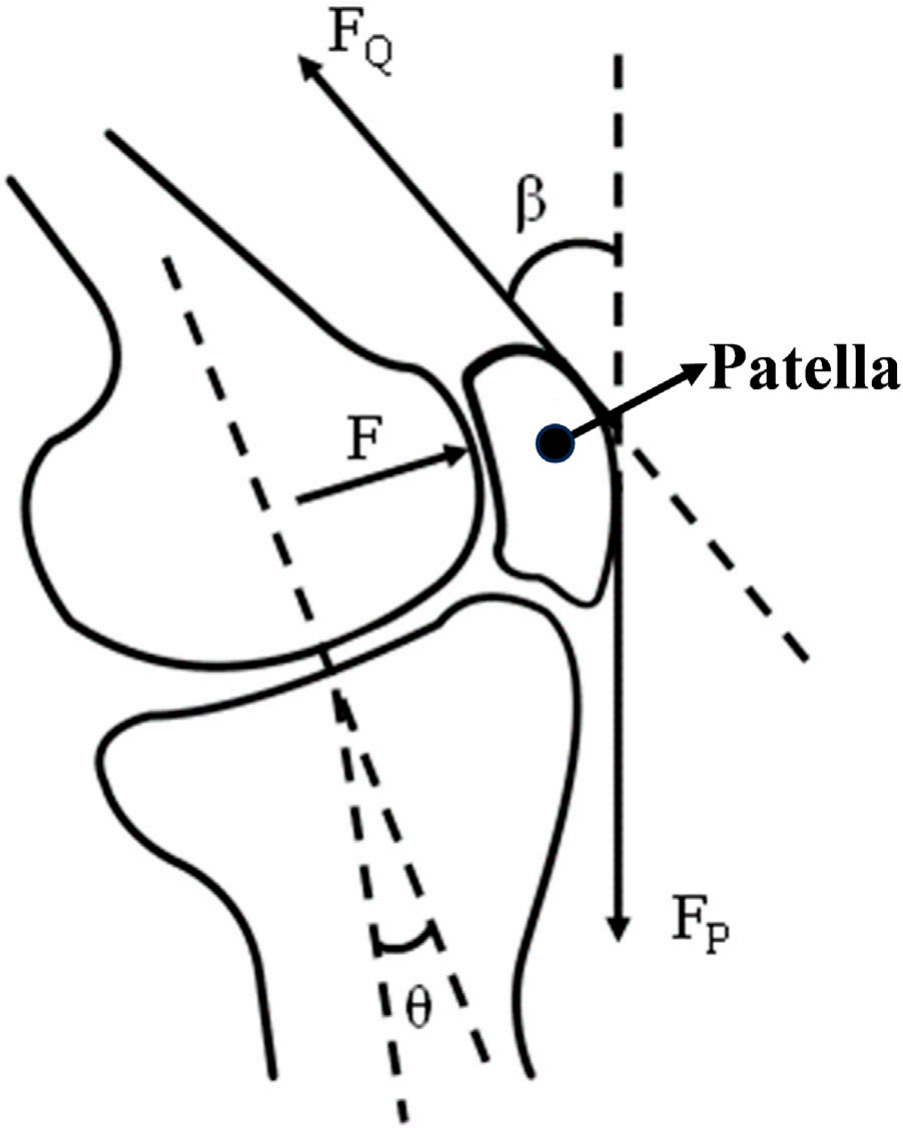
Schematic diagram of the patellofemoral joint isolator and associated angles.

In the equation, F_PF_ (N) represents the PFJF, and *β* (°) is the angle between the quadriceps muscle force line and the patellar ligament tension line (Figure 3).

PFJS calculation:

The contact area (mm^2^) between the patella and femur is a function of the sagittal plane knee joint angle θ (°). Huberti and Hayes (1984) were the first to identify a correlation between patellofemoral joint contact area and knee flexion angle. The patellofemoral joint contact area was found to be 2.6 ± 0.4 cm^2^, when the knee flexion angle was 20°, 3.1 ± 0.3 cm^2^ when the knee flexion angle was 30°, 3.9 ± 0.6 cm^2^ when the knee flexion angle was 60°, and 4.1 ± 1.2 cm^2^ when the knee flexion angle was 90°. Subsequent studies employed the data from the aforementioned investigation to ultimately derive a predictive equation for the patellofemoral joint contact area (Salem and Powers, 2001; Vannatta and Kernozek, 2015).

$$S_{PFCA}\left(\theta_{i}\right)=\left(0.0781\times {\theta_{i}}^{2}+0.6763\times \theta_{i}+151.75\right)$$

S_PFCA_ (*θ*_*i*_) represents the contact area (mm^2^) between the patella and the femur, from which the final PFJS is derived (Bressel, 2001; Vannatta and Kernozek, 2015).

$$P_{PFJS}\left(\theta_{i}\right)=F_{PF}\left(\theta_{i}\right)/S_{PFCA}\left(\theta_{i}\right)$$

In the equation *P*_*PFJS*_ represents the PFJS (MPa).

### 2.5 Statistical analysis

A one-way ANOVA with repeated measures was conducted using SPSS version 22.0 (SPSS Inc., IL, United States) software to assess the impact of different sensory integration tasks (BL, TIT, LIT, VIT) on the lower limb. The biomechanical characteristics of the lower limb in patients with PFP were analyzed. The dependent variables in this study were the three-dimensional angles, three-dimensional moments and power at the hip, knee and ankle joints at the moment of maximum PFJS, as well as step length, step width, cadence and foot forward angle. Pearson bivariate correlation analysis was employed to examine the relationship between gait characteristics (hip, knee, and ankle three-dimensional angles, three-dimensional moments, and power, in addition to step length, step width, cadence, and foot forward angle.) during the performance of the four interferences and the PFJS. The clinical evaluation criteria for correlation effect size were as follows: high correlation (|r| ≥ 0.50), moderate correlation (0.50 > |r| ≥ 0.30), and low correlation (0.30 > |r| ≥ 0.10). The criterion for statistical analysis was set as one-class error probability not exceeding 0.05. The present study was concerned with the effects of a multi-domain integration cognitive task on gait characteristics that appeared to be more subtle. To identify these subtle differences, the LSD method was employed as a *post hoc* multiple comparison technique. Accordingly, the conservative approach proposed by Bonferroni et al. was not employed to adjust the *p*-values. The effect size (η^2^) of the one-way ANOVA was calculated according to the following criteria: 0.01 ≦ η^2^ < 0.06 is classified as a low effect size, 0.06 ≦ η^2^ < 0.14 is considered a medium effect size, and η^2^ ≧ 0.14 is defined as a high effect size (Fritz et al., 2012).

## 3 Results

### 3.1 Impact of different sensory integration tasks on the biomechanical characteristics of the lower limb

**FIGURE 4:**
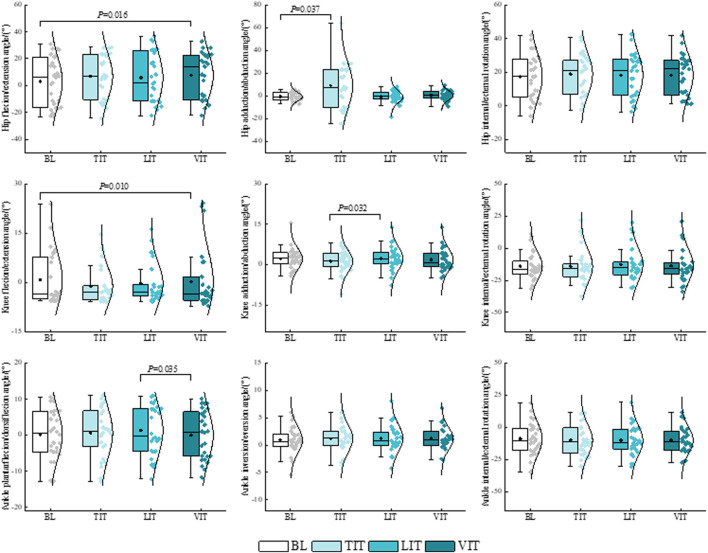
Impact of different sensory integration tasks on lower limb kinematic characteristics.

The results of the one-way repeated measures ANOVA demonstrated that patients with PFP exhibited a significantly greater hip flexion angle *P* = 0.016) and a significantly lesser knee flexion angle (*P* = 0.010) when walking and performing the VIT, compared to the BL condition. A notable increase in the hip adduction angle was observed during the execution of TIT (*P* = 0.037). A significantly greater knee adduction angle was observed during the performance of LIT compared to that observed during the performance of TIT (*P* = 0.032). The ankle plantarflexion angle was found to be significantly reduced during the performance of VIT in comparison to when performing LIT (*P* = 0.035) (Figure 4; Table 1).

**TABLE 1:** Impact of different sensory integration tasks on the biomechanical characteristics of the lower limb.

	BL (mean value)	TIT (mean value)	LIT (mean value)	VIT (mean value)	F value	η^2^
Hip flexion/extension angle/(°)	5.116	8.953	5.561	9.619	1.229	0.044
Hip adduction/abduction angle/(°)	−0.703	1.876	−0.733	0.446	2.745	0.092
Hip internal/external rotation angle/(°)	17.311	14.563	14.628	13.968	0.546	0.020
Hip flexion/extension moment/(N·mm/kg)	187.254	175.578	107.530	282.279	0.897	0.032
Hip adduction/abduction moment/(N·mm/kg)	221.159	243.859	269.427	144.468	1.112	0.040
Hip internal/external rotation moment/(N·mm/kg)	66.622	68.035	68.378	59.414	0.347	0.013
Hip power/(W/kg)	0.388	0.308	0.130	0.493	1.396	0.049
Knee flexion/extension angle/(°)	2.705	−0.684	1.984	1.364	2.721	0.092
Knee adduction/abduction angle/(°)	3.040	1.237	2.295	3.040	2.117	0.073
Knee internal/external rotation angle/(°)	−13.974	−14.030	−12.735	−13.590	0.501	0.018
Knee flexion/extension moment/(N·mm/kg)	−318.620	−354.652	−415.171	−371.747	1.416	0.050
Knee adduction/abduction moment/(N·mm/kg)	148.749	172.590	151.214	124.535	0.907	0.033
Knee internal/external rotation moment/(N·mm/kg)	73.935	88.405	84.564	80.779	0.971	0.035
Knee power/(W/kg)	0.263	0.182	0.348	0.225	0.487	0.018
Ankle plantarflexion/dorsiflexion angle/(°)	0.811	2.482	1.810	0.905	1.126	0.040
Ankle inversion/eversion angle/(°)	0.882	0.687	0.963	0.752	0.207	0.008
Ankle internal/external rotation angle/(°)	−7.985	−8.574	−7.766	−8.660	0.148	0.005
Ankle plantarflexion/dorsiflexion moment/(N·mm/kg)	578.255	533.471	627.887	480.083	1.550	0.054
Ankle inversion/eversion moment/(N·mm/kg)	−7.449	−1.909	0.101	0.239	0.353	0.013
Ankle internal/external rotation moment/(N·mm/kg)	79.371	76.304	85.846	69.771	1.143	0.041
Ankle power/(W/kg)	0.008	0.100	0.075	0.089	0.256	0.009
Step length/(m)	0.724	0.653	0.694	0.688	16.789	0.383
Step width/(m)	0.152	0.152	0.159	0.155	2.448	0.083
Cadence/(steps/min)	104.798	94.388	99.642	102.988	16.695	0.382
Foot progress angle/(°)	−4.628	−4.555	−5.514	−5.135	1.412	0.050

**FIGURE 5:**
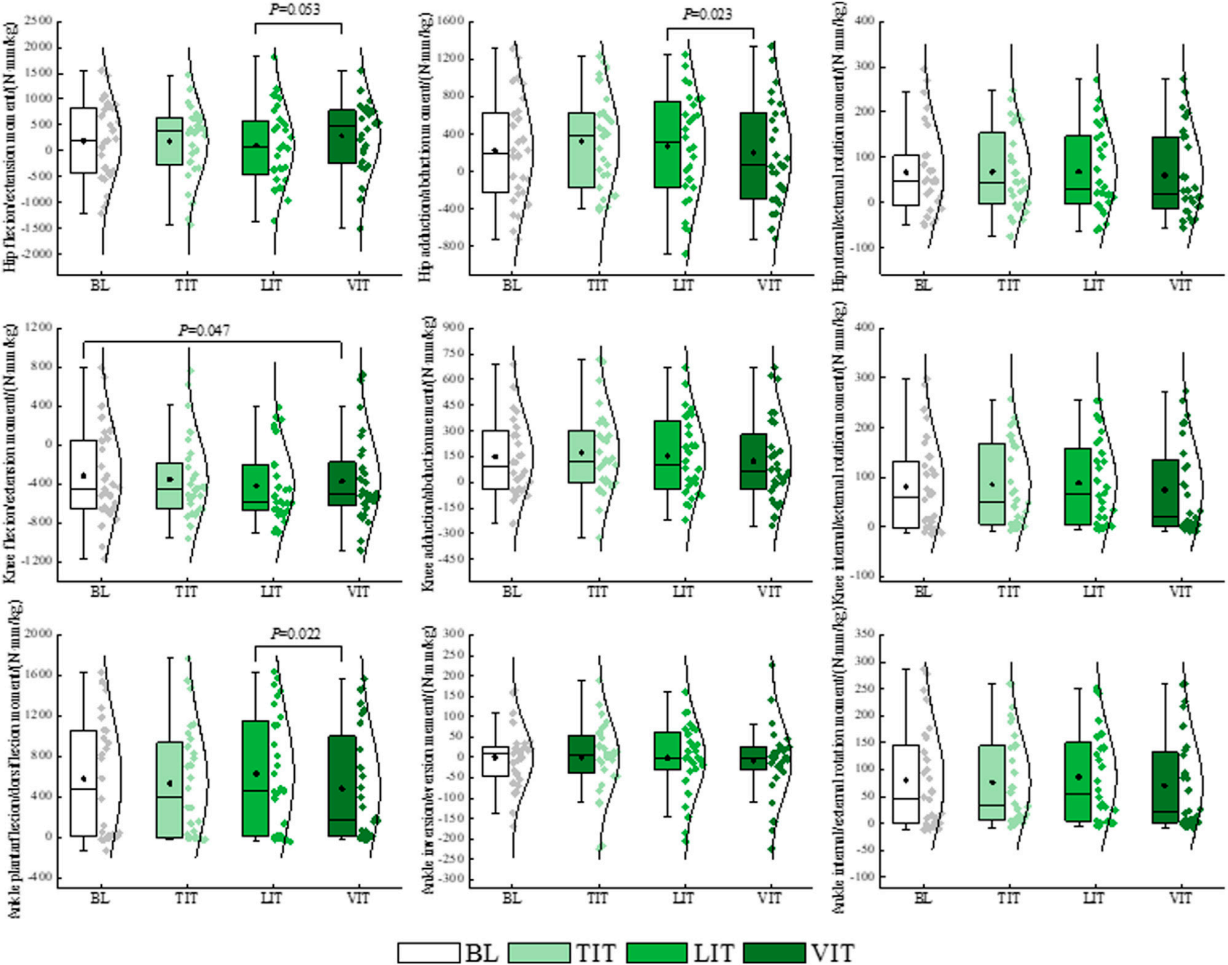
Impact of different sensory integration tasks on lower limb kinetic characteristics.

**FIGURE 6:**
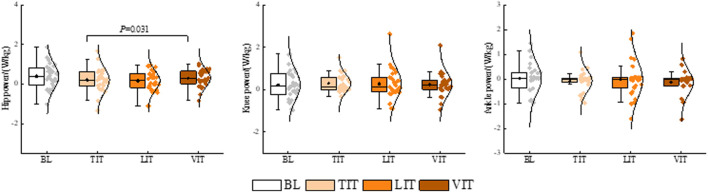
Impact of different sensory integration tasks on lower limb power.

The results of the one-way repeated measures ANOVA demonstrated that patients with PFP exhibited a significantly greater knee extension moment when walking and performing a VIT (*P* = 0.047), compared to the BL condition. A significantly greater hip power was observed during the performance of VIT compared to that observed during the performance of TIT (*P* = 0.031). A significantly greater hip flexion moment (*P* = 0.053), a significant reduction in hip adduction moment (*P* = 0.023), and a significant reduction in ankle plantarflexion moment (*P* = 0.022) were observed during the performance of VIT compared to that observed during the performance of LIT (Figures 5, 6; Table 1).

The one-way repeated measures ANOVA demonstrated that there were no statistically significant differences in PFJF and PFJS in patients with PFP with different sensory integration tasks (*P >* 0.05) (Table 2).

**TABLE 2:** Effects of different sensory integration tasks on PFJF and PFJS.

	BL	TIT	LIT	VIT
Patellofemoral joint force(N)	3129.867 ± 1325.845	3183.173 ± 984.370	3236.206 ± 1149.276	3217.740 ± 1023.327
Patellofemoral joint stress (MPa)	20.478 ± 9.146	20.922 ± 6.531	21.263 ± 7.730	21.175 ± 6.928

**FIGURE 7:**
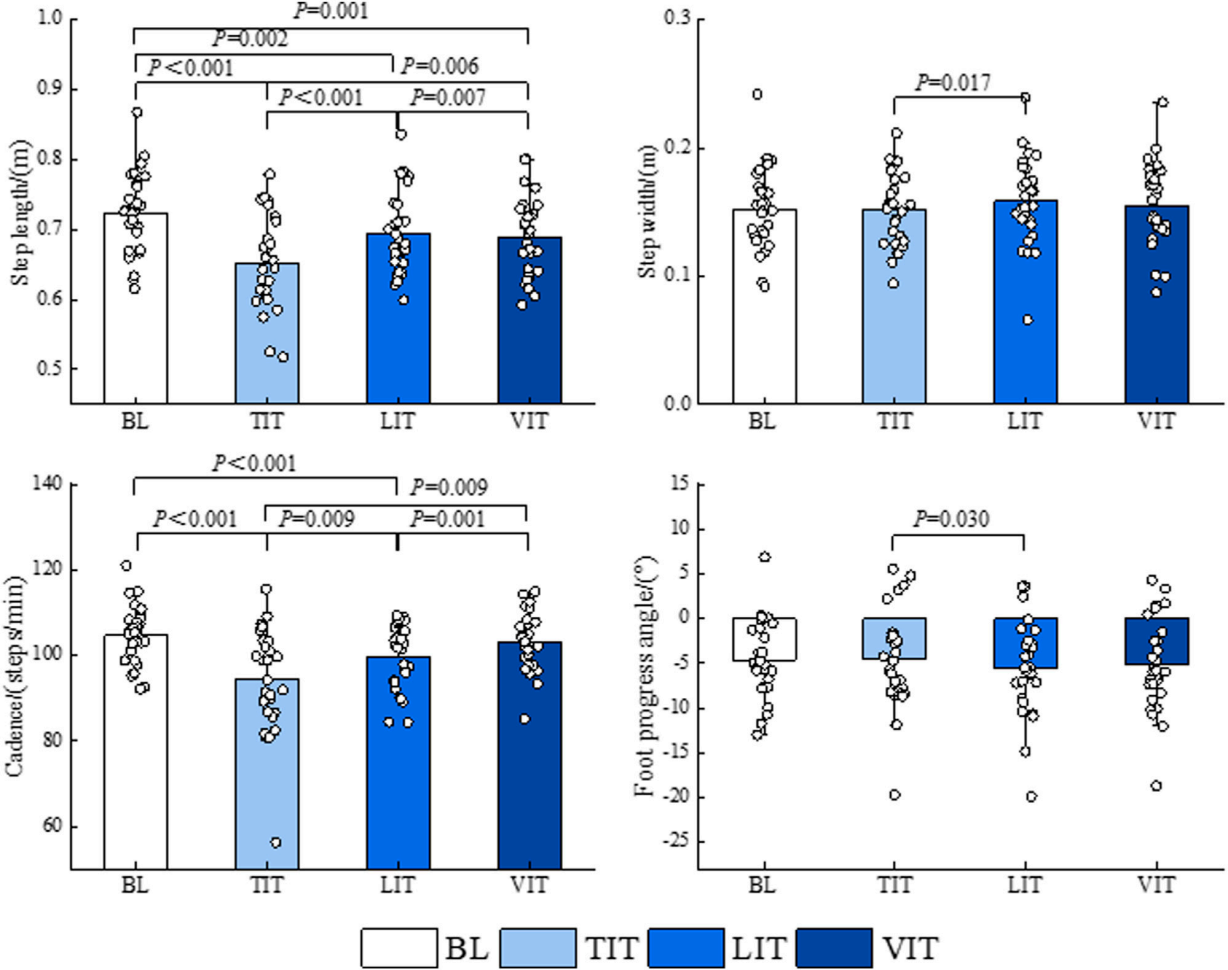
Impact of different sensory integration tasks on gait parameters.

The results of a one-way repeated measures ANOVA demonstrated that patients with PFP exhibited a 9.81% reduction in step length (*P <* 0.001) and a 10.01% reduction in cadence (*P <* 0.001) when walking and performing TIT compared to the BL condition. Furthermore, a 4.14% reduction in step length (*P* = 0.002) and a 4.92% reduction in cadence (*P <* 0.001) were observed when performing LIT while walking. On the other hand, a 4.97% reduction in step length occurred when performing VIT while walking (*P* = 0.001). Additionally, a 5.91% increase in step length (*P <* 0.001), a 4.40% increase in step width (*P* = 0.017), a 5.27% increase in cadence (*P* = 0.009), and a 17.39% increase in foot progress angle (*P* = 0.030) were observed when the LIT was performed compared to the TIT. The step length (*P* = 0.006) required to perform the VIT was 5.09% greater than that required for the TIT, while the cadence (*P* = 0.009) was 8.35% faster during VIT. A reduction of 0.86% in step length (*P* = 0.007) and an acceleration in cadence (*P* = 0.001) of 3.25% were observed when the VIT were performed in comparison to when LIT were performed (Figure 7; Table1).

### 3.2 Relationship between lower limb biomechanical characteristics and PFJS with different sensory integration tasks

Pearson correlation analysis revealed that, irrespective of interference type, that greater step length (BL: *r* = 0.494, TIT: *r* = 0.734, LIT: *r* = 0.827, VIT: *r* = 0.697) corresponds with higher PFJS (*P* < 0.05). Conversely, slower cadence (BL: *r* = −0.451, TIT: *r* = −0.650, LIT: *r* = −0.823, VIT: *r* = −0.854) and lesser knee flexion angle (BL: *r* = −0.502, TIT: *r* = −0.607, LIT: *r* = −0.447, VIT: *r* = −0.527) correspond with lesser PFJS (*P* < 0.05). In the LIT, that greater hip flexion angle (*r* = 0.388, *P* = 0.041) and knee extension moment (*r* = 0.468, *P* = 0.012) correspond with higher PFJS. The greater the hip flexion angle (*r* = 0.485, *P* = 0.009) and knee extension moment (*r* = 0.418, *P* = 0.027) correspond with higher PFJS in the VIT (Table 3).

**TABLE 3:** Relationship between the biomechanical characteristics of lower limb movement and PFJS for different sensory integration tasks at the moment of peak PFJS.

	BL		TIT		LIT		VIT	
	*r*	*P*	*r*	*P*	*r*	*P*	*r*	*P*
Step length/(m)	0.494	0.008	0.734	0.000	0.827	0.000	0.697	0.000
Step width/(m)	0.255	0.191	−0.186	0.343	−0.331	0.085	−0.214	0.275
Cadence/(m)	−0.451	0.016	−0.65	0.000	−0.823	0.000	−0.854	0.000
Hip flexion/extension angle/(°)	0.121	0.538	−0.094	0.636	0.388	0.041	0.485	0.009
Hip adduction/abduction angle/(°)	0.140	0.477	−0.230	0.224	−0.023	0.906	−0.017	0.932
Knee flexion/extension angle/(°)	−0.502	0.007	−0.607	0.001	−0.447	0.017	−0.527	0.004
Knee adduction/abduction angle/(°)	−0.286	0.141	0.206	0.132	0.183	0.144	−0.140	0.478
Knee internal/external rotation angle/(°)	−0.256	0.188	0.022	0.192	−0.072	0.715	−0.168	0.394
Ankle plantarflexion/dorsiflexion angle/(°)	−0.260	0.182	0.269	0.166	0.074	0.707	0.048	0.810
Hip flexion/extension moment/(N·mm/kg)	0.458	0.014	0.013	0.949	−0.045	0.821	0.082	0.679
Hip adduction/abduction moment/(N·mm/kg)	−0.029	0.882	0.179	0.363	0.125	0.273	0.327	0.090
Hip power/(W/kg)	−0.002	0.990	−0.046	0.816	0.068	0.730	−0.129	0.513
Knee flexion/extension moment/(N·mm/kg)	0.642	0.000	0.183	0.351	0.468	0.012	0.418	0.027
Ankle plantarflexion/dorsiflexion moment/(N·mm/kg)	−0.007	0.973	0.175	0.373	0.273	0.159	0.188	0.338

## 4 Discussion

The objective of this study was to examine the impact of different sensory integration tasks on the gait patterns of patients with PFP and to elucidate the relationship between these gait patterns and PFJS. The findings of the study corroborate the hypothesis that VIT exerts the most pronounced influence on gait pattern. The increased hip flexion angle and knee extension moment associated with VIT were found to be linked with an increased PFJS. Consequently, this study aimed to analyze metrics related to PFJS.

The findings of this study indicate that patients with PFP exhibit motor characteristics of reduced knee flexion angle when walking with a VIT. This can be explained by the attentional resource model, which suggests that if two or more tasks are performed simultaneously, the tasks interfere with each other (Al-Yahya et al., 2011; Hughes and Dai, 2023). In this study, patients with PFP were required to perform VIT while walking. A brain imaging study has demonstrated that motor control is dependent on prefrontal cortical activation and that brain regions controlling movement appear to be interconnected with neural networks for higher cognitive functions (Al-Yahya et al., 2011). Therefore, when a VIT competes with motor control for shared neural networks, individuals may exhibit alterations in their motor characteristics (Al-Yahya et al., 2011). As previously stated, the VIT in this study encompassed a range of activities, including vision and walking, necessitating that participants prioritize accuracy in the visual task. The input of a substantial quantity of visual data consequently requires a greater allocation of cognitive resources, which may impair the brain’s capacity to precisely regulate joint movements. This can result in the inability of the patient to accurately control the degree of knee flexion during ambulation, potentially manifesting as gait characteristics such as a diminished knee flexion angle. The results of the present study demonstrated that a decrease in the knee flexion angle was associated with an increase in PFJS. This may be attributed to the fact that landing with the knee in an upright position does not effectively cushion the ground reaction forces. The application of higher ground reaction forces to the entire lower limb results in an increased internal load on the knee joint, which in turn induces stress on the patellofemoral joint, leading to the development of PFP (Hughes and Dai, 2023; Willy and Meira, 2016). The other rationale can be attributed to the PFJS calculation model employed in this study. A reduction in the knee flexion angle will result in a diminution of the effective force arm of the quadriceps muscle and the contact area of the patellofemoral joint, which will consequently lead to an augmentation in the quadriceps muscle force, and ultimately, an increase in the patellofemoral joint force and PFJS.

The findings of the present study indicate that patients with PFP exhibit greater knee extension moments when walking while performing VIT. Furthermore, the observed increase in knee extension moment is associated with augmented PFJS. This finding is consistent with that of a study by Hughes and Dai (2023). In order to complete the VIT, patients with PFP must be able to recognize and read the text displayed on the computer monitor in front of them, and to ensure that their reading is accurate. This aspect may require greater attentional resources, which could consequently impact performance on walking tasks (Al-Yahya et al., 2011; Hughes and Dai, 2023). Another aspect, VIT may result in a reduction in the patient’s visual input during normal ambulation. Previous studies have indicated that the lack of such feedback during walking leads to suboptimal foot placement, which in turn reduces the ability of the arch and ankle to cushion against ground reaction forces (Shoja et al., 2024). The remaining ground reaction forces continue to be compensated upwards to the knee joint (Brown et al., 2014; Shoja et al., 2024). At this juncture, the knee joint is required to sustain a considerable extension moment to facilitate more efficient landing cushioning and avert joint collapse (Brown et al., 2014; Shoja et al., 2024). However, excessive knee extension moments can result in abnormal patellar trajectories and increased patellofemoral joint stress. Previous studies have indicated that patellofemoral joint stress increases by 3.9 MPa when the knee extension moment reaches 240 Nm (Takabayashi et al., 2019).

The findings of the study indicated that visual perturbations resulted in an augmented hip flexion angle during ambulation in patients with PFP. Previous studies have demonstrated that trunk control requires greater attentional resources and is susceptible to interference from additional cognitive load. Consequently, when an individual is exposed to visual perturbations, a significant amount of attentional resources are allocated (Eckstein, 2017), resulting in a reduction in the brain’s control of the trunk, necessitating the individual to utilize compensatory strategies, such as increased hip flexion, in order to maintain trunk stability (Logan et al., 2010; Nevisipour et al., 2023). Additionally, studies from a neuromuscular perspective offer insight into the above findings. It can be observed that humans typically adopt a gait in which the front leg serves to cushion the impact and the back leg completes the pedal and stretch. This process is susceptible to generating “imbalance moments,” which can result in the body tilting forward or backward (Logan et al., 2010). In the absence of any external disturbances, the brain is capable of regulating this “imbalance moment” by activating the peripatellar muscles (Logan et al., 2010). However, when an individual performs VIT while walking, the visual perception of other environmental information decreases, and the brain’s feedback regulation of body balance decreases. Consequently, the peripatellar muscles cannot be activated in a timely and rapid manner to overcome the aforementioned “imbalance moment.” At this juncture, the patient’s trunk appears to exhibit a pronounced swing, predominantly in the form of an elevated hip flexion angle (Logan et al., 2010). It is crucial to highlight that this study identified a correlation between an increased hip flexion angle and elevated PFJS. Saha et al. (2008) demonstrated that an elevated hip flexion angle results in a forward shift of the body’s center of gravity, which in turn leads to an increase in hip extension moments. Furthermore, an increased hip flexion angle indirectly increases the level of activation of the hamstrings and gastrocnemius muscles, thereby disrupting the normal flexor-extensor co-contraction pattern of the knee. This ultimately results in elevated PFJS (Alghamdi and Preece, 2020).

The present study demonstrated that walking while performing both LIT and TIT resulted in a reduction in step frequency in patients with PFP. It has been demonstrated that the auditory system plays a pivotal role in maintaining the dynamic stability of an individual during movement. Furthermore, auditory cognitive perturbations utilizes a portion of the listening information resources, which results in a reduction in the brain’s feedback regulation of the body’s postural balance. This is primarily manifested as gait instability (Hallemans et al., 2010; Iosa et al., 2012; Maheu et al., 2017). At this juncture, the individual attempts to reduce the walking speed by decreasing the stride frequency, thereby maintaining dynamic stability during the gait cycle (Saucedo and Yang, 2017). It has also been observed that the performance of TIT while walking increases the attentional demands of combinatorial tasks. Consequently, the brain is unable to allocate all of its attention to maintaining walking homeostasis. In such circumstances, the brain often employs protective gait strategies, such as a reduction in cadence, in order to prevent gait instability (Oates et al., 2017).

The results of this study demonstrate that TIT exerts a more pronounced influence on cadence than LIT. This can be attributed to the intrinsic mechanism that governs human gait. When humans engage in walking tasks, they must integrate information from multiple sensory systems, including somatosensory, visual, and auditory centers, to facilitate gait adjustments in complex environments and to maintain a stable body posture. Nevertheless, the manner in which humans utilize the aforementioned sources of information differs. In general, 70% of gait regulation information is derived from tactile or proprioceptive sources (Horak, 2006). However, the occupation of the brain’s somatosensory information resources by TIT will result in a significant reduction in the amount of effective sensory information input, leading to a substantial shift in the center of gravity of the individual and even the loss of balance. It is challenging for the human body to maintain a normal walking frequency when in a state of imbalance. Nevertheless, Van Hooren et al. (2024) observed that a reduction in cadence may result in elevated knee joint loading and an augmented PFJS, and the findings of the correlation analyses in this study further substantiate this conclusion. The underlying mechanism may be that a reduction in cadence increases the peak internal moment of the knee extension, which suggests that the instantaneous quadriceps muscle strength is significantly higher during walking. It has been demonstrated that the additional contraction force generated by the quadriceps muscle increases the internal pressure of the knee joint, thereby accelerating the deterioration of the cartilage and causing PFP (Hart et al., 2020).

The findings of this study indicate that walking in a dual-task state results in a reduction in step length in patients with PFP. This result is consistent with the findings of previous studies in this area (Lau et al., 2021). Lau et al. (2021) study demonstrated that the regulation of gait during human walking is actually based on the perceptual memory of the foot and body. In particular, when walking in a dual-task state, the brain has a greater demand for the quantity of sensory information input, which results in a more precise integration of motor information and adjustment of the motor program (Lau et al., 2021). However, each dual-task model in this study necessitated the utilization of the participants’ mental imagery and perceptual memory (Cinar et al., 2021), which frequently resulted in a reduction in cortical sensory input. This, in turn, led to participants experiencing impaired balance and being compelled to utilize shorter step lengths in order to complete the walking task (Lau et al., 2021).

Furthermore, the results of this study indicate that TIT has a significantly greater impact on step length in patients with PFP than LIT and VIT. Previous research has explored this phenomenon in detail and found that the dual-task cost of tactile perturbations is higher compared to visual and listening perturbations (Nguyen et al., 2020). When performing both the tactile and walking tasks simultaneously, the majority of attentional resources will be directed towards the tactile stimulus sequence, which will severely inhibit the sensorimotor control of the walking task. This will result in the occurrence of gait abnormalities, such as a reduction in stride length, with a high probability (Ishida et al., 2020; Nguyen et al., 2020). It is important to note that a reduction in step length may have served as a protective mechanism for patients with PFP. Research has demonstrated that a reduction in step length significantly reduces knee energy absorption and PFJF (Lenhart et al., 2014). Additionally, Willson et al. (2016) demonstrated that a 10% reduction in step length resulted in a 15%–20% reduction in PFJF compared to self-selected step length, which was found to reduce the risk of recurrent PFP.

The findings of the study indicated that the execution of diverse sensory integration tasks by patients with PFP during ambulation exerted a discernible influence on the characteristics of their gait. The VIT had the most significant effect on the gait of patients with PFP. For instance, the VIT resulted in increased hip flexion angle, knee extension moment, and decreased knee flexion angle and stride frequency. These changes may contribute to an increase in PFJS. In light of these findings, it can be posited that patients may potentially develop resistance to visual perturbations during ambulation through the implementation of low-load visual multitasking training. This could serve to mitigate the deleterious effects of visual disturbances on gait, whilst simultaneously enhancing the capacity of patients to maintain optimal gait patterns. Wong et al. (2023) observed that following the completion of dual-task training, patients demonstrated enhanced step count and step length, along with improved attention and executive function, when undertaking a multi-domain integration task.

It should be noted that this study is not without limitations. The present study did not include female patients with PFP, and no comparison was made between genders. However, previous studies have indicated that there are significant differences in the pathogenesis of PFP in male patients compared to female patients (Boling et al., 2019). For example, it has been proposed that excessive hip adduction may have a deleterious impact on the patellofemoral joint (Powers, 2010). However, Nakagawa et al. (2012) observed that female patients with PFP exhibited a diminished capacity to generate hip abduction moments relative to males. It is therefore imperative that the gender factor be taken into account when designing or developing prevention or rehabilitation programmers related to PFP. It is imperative that future studies employ female patients with PFP as subjects if they intend to generalize the findings of this study to female patients with PFP. A deeper understanding of the mechanisms underlying their responses to different sensory multi-domain integration tasks would facilitate the development of more targeted recommendations. Second, it is possible that the quadriceps force arm calculation model employed in this study may have yielded results that are over-estimated. However, it can be reasonably concluded that this potential error falls within acceptable limits. It should be noted that the present study was not corrected for *p*-values in order to identify the subtle effects of a multi-domain integration cognitive task on gait. This may have increased the probability of Type I errors. Nevertheless, the fact that the majority of the findings from the present study align with those of previous studies indicates that the results were robust and reliable, despite the lack of correction for the *p*-value. Finally, the participants in this study completed the test in a pain-free state. The current state of research has yet to elucidate whether the underlying mechanism of pain-altered gait is a change in mechanical dynamics resulting from the pain itself, or whether pain occupies cognitive resources, which in turn gives rise to alterations in gait patterns. This is a topic meriting further investigation and provides a potential avenue for future research. It is anticipated that future studies will provide a more comprehensive understanding of the underlying mechanisms associated with pain-altered gait. The absence of a control group in this study precludes the possibility of determining whether the observed results are specific to patients with PFP. Further research could investigate the differences between PFP patients and non-PFP patients, with a view to developing new strategies for the prevention and treatment of PFP in the future. It should be noted that, walking is less susceptible to PFP than running or walking up and down stairs. However, given the prevalence of walking, this study focused exclusively on this particular movement. Further research could examine more challenging movement tasks, such as running, stair climbing and others, in order to provide additional insights for the prevention and treatment of PFP.

## 5 Conclusion

The characteristics of lower limb movement are altered in patients with PFP when they are subjected to disturbances in the form of tactile, listening, or visual perturbation while walking. Of these, visual perturbations have the greatest effect on lower limb movement patterns. During VIT, patients with PFP exhibited motor characteristics that may increase PFJS and the risk of recurrence of PFP. These characteristics included increased hip flexion angle, decreased knee flexion angle, increased knee extension moment, decreased step length, and decreased cadence. Among these factors, increased hip flexion angle and knee extension moments, decreased knee flexion angle, and cadence may increase PFJS and thereby increase the risk of PFP recurrence. In contrast, the cadence of patients with PFP was significantly reduced during the performance of TIT and LIT. This may be the causal factor for the increased PFJS in these two cognitive states. It is recommended that patients with PFP avoid the occupation of cognitive resources by visual perturbation while walking. It is recommended that dual-task training and gait feedback training be undertaken if necessary, as these have been shown to improve gait stability. This is beneficial in reducing the risk of PFP recurrence, as they have been demonstrated to ameliorate the detrimental effects of multidomain integration tasks on gait.

## Data Availability

The original contributions presented in the study are included in the article/supplementary material, further inquiries can be directed to the corresponding author.
